# Increased CD34 in pancreatic islet negatively predict islet β-cell decrease in type1 diabetes model

**DOI:** 10.3389/fphys.2022.1032774

**Published:** 2022-11-18

**Authors:** Shichen Huang, Zhiyuan Li, Yuhan Sun, Baiyi Chen, Yuxin Jiang, Feng Hong

**Affiliations:** ^1^ School of Preclinical Medicine, Wannan Medical College, Wuhu, China; ^2^ School of Pharmacy, Wannan Medical College, Wuhu, China; ^3^ Department of Population and Public Health Sciences, Keck School of Medicine of University of Southern CA, Los Angeles, CA, United States; ^4^ Jiaxing Key Laboratory of Virus-Related Infectious Diseases, The First Hospital of Jiaxing City, Jiaxing University, Jiaxing, China; ^5^ The Brain Cognition and Brain Disease Institute, Shenzhen Institute of Advanced Technology, Chinese Academy of Sciences, Shenzhen, China

**Keywords:** CD34, islet β-cell, β-cell marker, β-cell transdifferentiation, type 1 diabetes

## Abstract

Islet β-cell biomarkers can reflect changes in the number and function of islet β-cells in the prediabetes or early diabetes stage. CD34 is a commonly used stem cell biomarker; however, its expression and function in pancreatic islets remain unclear. In the present study, double immunofluorescence staining, proteomic bioinformatics analysis, and correlation analysis were used to explore the potential of CD34 as an islet β-cell biomarker. Bioinformatics analysis revealed that the amino acid sequence of CD34 was conserved among multiple species and abundantly expressed on mouse and human pancreatic tissues. Immunofluorescence demonstrated that in the control rat pancreas, CD34 was expressed on glucagon-labeled islet α-cells but not on insulin-labeled islet β-cells. Furthermore, the proportion of CD34-positive cells, which were also positive for glucagon, was significantly increased in alloxan-induced diabetes models. Statistical analysis revealed that the expression of CD34 was negatively correlated with the number of insulin-labeled islet β-cells during diabetes progression in dose-dependent fashion in alloxan-induced diabetes models. Furthermore, the results suggested that the transdifferentiation of islet β-cells into islet α-cells may occur in the process of diabetes. Thus, the present study demonstrated that CD34 is expressed on islet α-cells, and its number is linearly and negatively correlated with the number of islet β-cells, suggesting that CD34 can be used as a prospective biomarker for islet β-cells in the early diagnosis of diabetes. The study also suggests the transformation of β-cells to α-cells in diabetes which provide a potential to be applied towards diabetes mechanism research.

## 1 Introduction

The pancreatic islets primarily comprise glucagon (GLU)-secreting α-cells, insulin (INS)-secreting β-cells, somatostatin-secreting δ-cells, pancreatic polypeptide (PP)-secreting PP cells, and ghrelin-secreting ε cells (Pan and Wright, 2011). Diabetes is characterized by progressive β-cell failure (Cnop et al., 2005). Furthermore, the decrease and dysfunction of β-cells are the key factors that lead to diabetes mellitus (D'Addio et al., 2022; Veres et al., 2019).

The early diagnosis of diabetes is critical for its treatment and prognosis. A fasting blood glucose of >7.00 mmol/L is often considered the preliminary diagnostic standard of diabetes in the clinic; however, by the time hyperglycemia is detected, most islet β-cells have already been irreversibly damaged. The monitoring of changes in the β-cells in pancreatic islets is required for the early diagnosis and treatment of diabetes. At present, the common diagnostic methods, including the diagnostic index of type 1 diabetes 60, oral glucose tolerance test, glycosylated hemoglobin, and some biomarkers for the risk prediction and clinical diagnosis of β-cell autoantigen such as Glutamate Decarboxylase 65 (Schlosser et al., 2008), C-peptide (Sims et al., 2019), and proinsulin (Titova et al., 2020), always diagnose diabetes after most β-cells are injured. Therefore, it is important to explore a biomarker for monitoring early β-cell changes in the clinical diagnosis and intervention of diabetes (Sims et al., 2018).

The role of leukocyte differentiation antigen [cluster of differentiation (CD)] in islet endocrine function has recently been acknowledged. In the progression of autoimmune diabetes, islet β-cells and others endocrine cells change with the increase in CD45-labeled leukocyte infiltration (Plesner et al., 2014). CD49a was utilized as a surface marker of β-cell population in the magnetic sorting of cultured β-cells (Veres et al., 2019). The CD44 variant CD44V inhibits the INS secretion of islet β-cells by inhibiting amino acid uptake (Kobayashi et al., 2018). As an important CD molecule, CD34 plays an important role in cell adhesion, signal transduction, proliferation, differentiation, and other processes (Deterding et al., 2011). CD34 is primarily expressed on hematopoietic stem and progenitor cells (Nielsen and McNagny, 2008; Sidney et al., 2014) as well as on non-hematopoietic cells such as mesenchymal stromal cells (Lin et al., 2012), muscle satellite cells (Beauchamp et al., 2000), epithelial cells (Hsu et al., 2011), and mast cells (Metcalfe, 2008). CD34 is widely present on the surface of stem/progenitor cells, and its antigenic structure, which is often used as a stem cell/progenitor cell biomarker to screen and isolate stem/progenitor cells with strong regeneration potential, is stable (Sidney et al., 2014; Tasev et al., 2016). It has been reported that CD34 is expressed on fetal bovine islet α-cells, and CD34 reactive cells contribute to the formation of α-cell groups in the early development of bovine embryos (Merkwitz et al., 2011). However, the expression and function of CD34 in adult rat islets are still unclear. Considering the conserved expression of CD34 among multiple species (Deterding et al., 2011), CD34 may play an important role in islet endocrine function, which may be used as a biomarker for islet cells, just as it does in other cells.

Islet β-cell apoptosis is the major form of β-cell failure in diabetes (Cnop et al., 2005; Moin and Butler, 2019). In recent years, attention has been paid to the relationship between β-cell dedifferentiation and trans-differentiation in diabetes (Moin and Butler, 2019), in which the percentage of INS-positive cells in the islet structure is decreased and the corresponding percentage of GLU-positive cells is increased (Titova et al., 2020). The β-cells lose the characteristics of mature cells and subsequently lose the ability to secrete INS because of gene regulation (Bensellam et al., 2018; Moin and Butler, 2019). These reports suggest that islet β-cell transdifferentiation is involved in islet β-cell reduction and dysfunction together with β-cell apoptosis.

In the present study, the potential of CD34 as an islet β-cell biomarker and as a β-cell transdifferentiation biomarker in diabetes was explored.

## 2 Materials and methods

### 2.1 Experimental animals and groups

Forty male Sprague Dawley rats (8–10 weeks old) were purchased from the same batch and numbered according to the ascending order of body weight. All rats were randomly classified into four groups: control group (*n* = 10), 100 mg/kg alloxan group (*n* = 10), 120 mg/kg alloxan group (*n* = 10), and 150 mg/kg alloxan group (*n* = 10). The rats in each group were fed adaptively for 1 week under the same environmental conditions of room temperature (22°C–24°C), light/dark cycle of 12 h, and free access to food and water. All study experiments were approved by the Animal Experimental Ethics Committee of Wannan Medical College following the Administration Regulations on the Use of Laboratory Animals in Anhui Municipality.

### 2.2 Type 1 diabetes model rats

Rats in each group were fasted for 16 h and then injected with a relative dose of alloxan solution (Table 1) according to their body weight. The food intake was normalized 2 h after administration. The blood glucose of rats in each group was measured at 48 h and 72 h after administration. The success of the diabetes model was identified *via* the following parameters: fasting blood glucose >11.1 mmol/L or random blood glucose >16.7 mmol/L. Rats with hyperglycemia (>33.3 mmol/L) at 48 h were decapitated, and their pancreas was quickly separated and collected; for the other rats, these processes were performed at 72 h (Chen et al., 2014; Hong et al., 2014).

**TABLE 1:** Main reagents used in this research.

Main reagent	Code	Company	Dilution/Dose
Alloxan	A7413-25G	Sigma-Aldrich	100 mg/kg; 120 mg/kg; 150 mg/kg
SAKURA Tissue-Tek O.C.T. Compound	4583	SAKURA	
Triton X-100	1139ML100	BioFroxx	0.3%
Normal Equine Serum	SL042	Solarbio	5%
DAPI	D9542-5 MG	Sigma-Aldrich	1:1000

#### 2.3 Pancreatic tissue management

The rats were sacrificed under anesthesia, and their pancreas were collected immediately and fixed in 4% paraformaldehyde solution at 4°C for 24 h. Subsequently, all pancreatic tissues were gradient-dehydrated in 15% and 30% sucrose solutions and then embedded in optimal cutting temperature solution (Table 1). The tissues were then cut into 6–8-μm sections at −20°C.

#### 2.4 Immunofluorescence

The pancreatic sections were washed with phosphate-buffered saline containing 0.3% TritonX-100 (PBST) (Table 1) and then incubated in 5% horse serum for 30 min (Table 1) to block the nonspecific binding of antibodies. Later, the sections were incubated with two different primary antibodies (Table 2) at 4°C overnight. As for the negative control experiment, PBST was used to replace the corresponding primary antibody in the incubation stage of the primary antibody, and the other steps were the same as above, in addition, in the single label staining control experiment, the incubation step of the primary antibody involves only one antibody. After incubation, the primary antibody was washed off using PBST, and the sections were incubated with the corresponding fluorescent secondary antibodies (Table 3) for 2 h at room temperature. The tissue sections were counterstained with 4-diamino 6-diamino-2-phenylindole dihydrochloride (DAPI) (Table 2) for 10 min and then washed with PBST. The tissue sections were observed, and the IF staining images were processed using a laser confocal microscope (Leica, Germany) and corresponding software.

**TABLE 2:** Primary antibodies used in this research.

Primary antibody	Code	Company	Host species	Dilution
Anti-CD34 antibody	ab185732	Abcam	Rabbit	1:400
Insulin Antibody	I2018	Sigma-Aldrich	Mouse	1:1000
Glucagon (N-17) antibody	sc-7780	Santa Cruz	Goat	1:400

**TABLE 3:** Secondary antibody used in this research.

Secondary antibody	Code	Company	Host species	Dilution
Alexa Fluor® 488 AffiniPure Donkey Anti-Rabbit IgG (H+L)	711–545-152	Jackson ImmunoResearch	Donkey	1:1000
Alexa Fluor® 594 AffiniPure Donkey Anti-Mouse IgG (H+L)	715–585-150	Jackson ImmunoResearch	Donkey	1:1000
Donkey Anti-Goat IgG H&L (Alexa Fluor® 594)	ab150132	Abcam	Donkey	1:1000

### 2.5 Fluorescence image analysis

The fluorescence images were preprocessed using LASAF software and automatically combined into a composite image containing red (INS positive area or GLU positive area), green (CD34 positive area), and blue (nucleus). Using the software Image Pro Plus (Table 4) to further process all the composite fluorescence images, the entire islet region was delineated using the Irregular AOI tool, and the red fluorescence, green fluorescence, and the entire islet areas in each islet region were calculated. The proportion of each antigen-labeled region to the islet area was calculated using the following formula: the area of the labeled area was divided by the area of the entire islet area. Furthermore, DAPI fluorescence was used to determine the number of antigen-labeled cells in the islet as well as the total number of islet cells by manual counting, and the proportion of each antigen-labeled cell to the total number of islet cells was calculated as follows: the number of cells of the same type was divided by the total number of cells in the islets.

**TABLE 4:** The software used in the study.

Software	Official address
Image Pro Plus	https://www.mediacy.com/imageproplus
MEGA 11	https://www.megasoftware.net/dload_win_gui
TBtools	https://github.com/CJ-Chen/TBtools/releases
GraphPad prism 9.0	https://www.graphpad-prism.cn/

#### 2.6 Correlation analysis

From the islet sample data, the expression data of CD34 and INS were screened in each islet and imported into GraphPad Prism 9.0 to draw a scatter plot. The “Correlation” algorithm command in the “Analyze” command function group of the software was used to analyze correlation between data, determine the p-value and the correlation coefficient r, and analyze differences. At the same time, the linear regression analysis of the sample scattered data was performed using the “linear regression” algorithm.

### 2.7 CD34 amino acid sequence alignment and phylogenetic tree construction of multiple species

The CD34 amino acid sequences of the representative species of primates, rabbits, odd hooves, cloven hooves, rodents, carnivores, and others were retrieved from the NCBI (https://www.ncbi.nlm.nih.gov/) database. The obtained amino acid sequences were preliminarily sorted out, and the sequence data were compared and analyzed using the software MEGA 11, and the CD34 phylogenetic tree was constructed using the “PHYLOGENY” command and the “neighbor-joining” method.

### 2.8 Acquisition and analysis of CD34 expression data in human and mouse tissues and organs

Proteomics DB (https://www.proteomicsdb.org) (Wilhelm et al., 2014; Schmidt et al., 2017) was used to screen the expression data of CD34 in human (*Homo sapiens*) and mouse (*Mus musculus*) tissues and organs. The data were analyzed and sorted, and the average value of CD34 expression in each tissue or organ was determined. The sorted data were imported into the software TBtools (Chen et al., 2020), and the “HeatMap” command in the “Graphics” function component was used to draw the heat map; the color of the heat map corresponded to the expression value.

### 2.9 Data processing and statistical analyses

The pancreatic tissue samples of five rats were randomly selected from each group *via* the random number table method for experiment and analysis. GraphPad Prism 9.0 was used for data processing and graph plotting; the data were analyzed using independent sample *t*-test, paired sample *t*-test, and one-way analysis of variance as well as linear regression analysis and correlation analysis. Before the data analysis, the statistical software is used to test the normality of the data, and the parameter test can be used for the follow-up statistical analysis of the data in accordance with the normal distribution. The data that do not conform to the normality test first judge whether there are outliers according to their distribution box diagram, and if there are outliers, Q-test is further used to analyze whether the corresponding outliers can be eliminated. If the data set that can be eliminated can be re-tested to conform to the normal distribution, the parameter test can be used for follow-up statistical analysis, otherwise nonparametric test should be used for statistical analysis. All data are presented as mean ± standard error, and *p* < 0.05 indicated statistical significance.

## 3 Results

### 3.1 Bioinformatics analysis revealed CD34 expression in the pancreas

**FIGURE 1:**
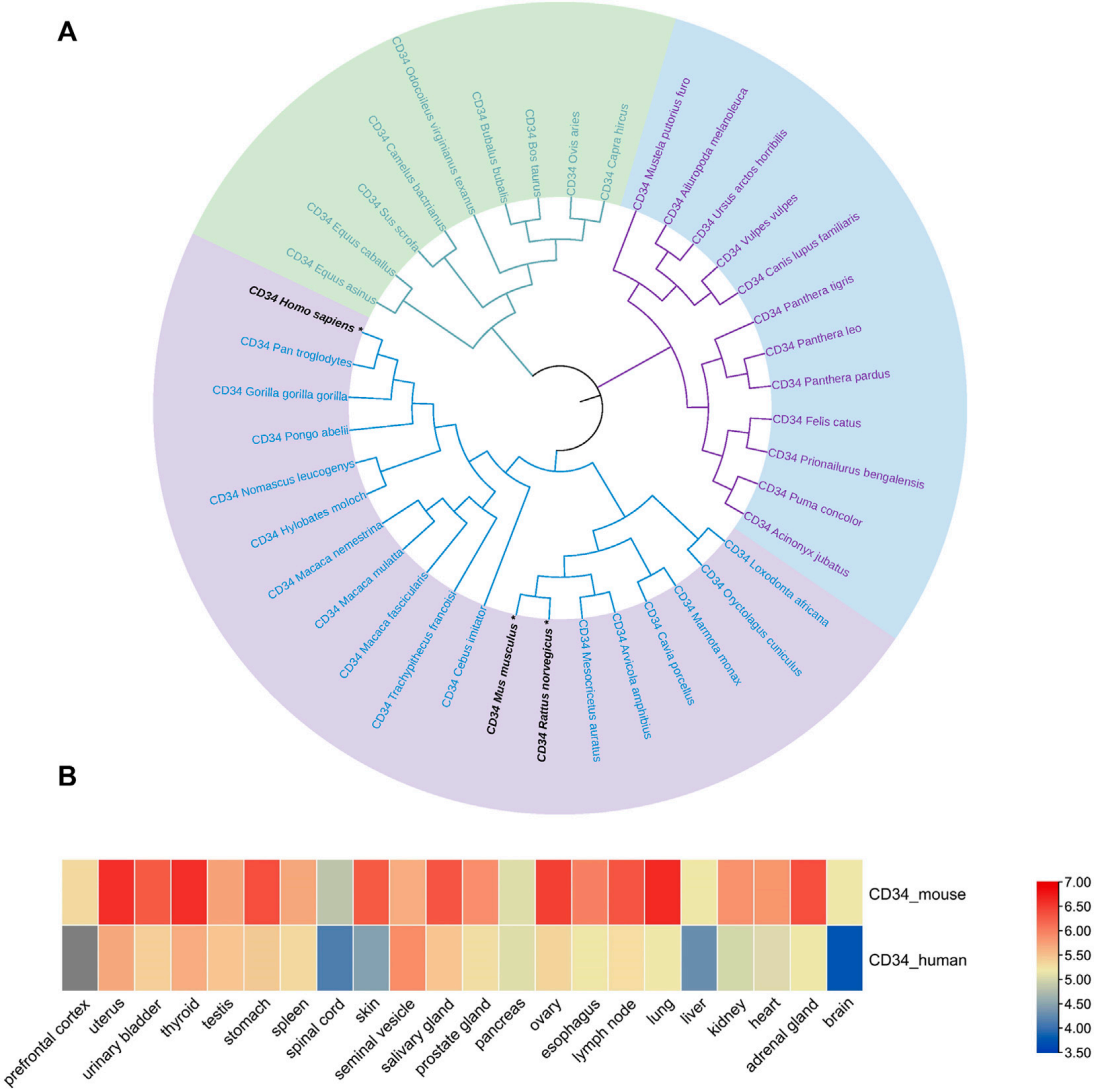
Genetic relationship of the CD34 sequence among species and its expression in the organisms. Figure **(A)** the evolutionary relationship of CD34 among species. Figure **(B)** the expression of CD34 in human and mouse tissues and organs.

First, the amino acid sequence data of multiple species were retrieved from the NCBI database, and the phylogenetic tree of the CD34 family members was constructed using MEGA 11. The analysis revealed that (Figure 1A) CD34 was conserved among the analyzed species. In addition, the CD34 sequences of rats, mice, and humans were in the same large branch of phylogenetic tree (Figure 1A), suggesting that rats or mice can be considered as research objects when studying CD34. ProteomicsDB was utilized to evaluate the expression of CD34 on various tissues and organs of humans (Homo) and mouse (*Mus musculus*). Data analysis using TBtools revealed that CD34 was expressed to a certain extent in both human and mouse pancreas (Figure 1B). Thus, the expression of CD34 in the pancreas and its relationship with islet endocrine cells were subsequently explored.

### 3.2 Consistent expression of CD34 in different part of rat islets

**FIGURE 2:**
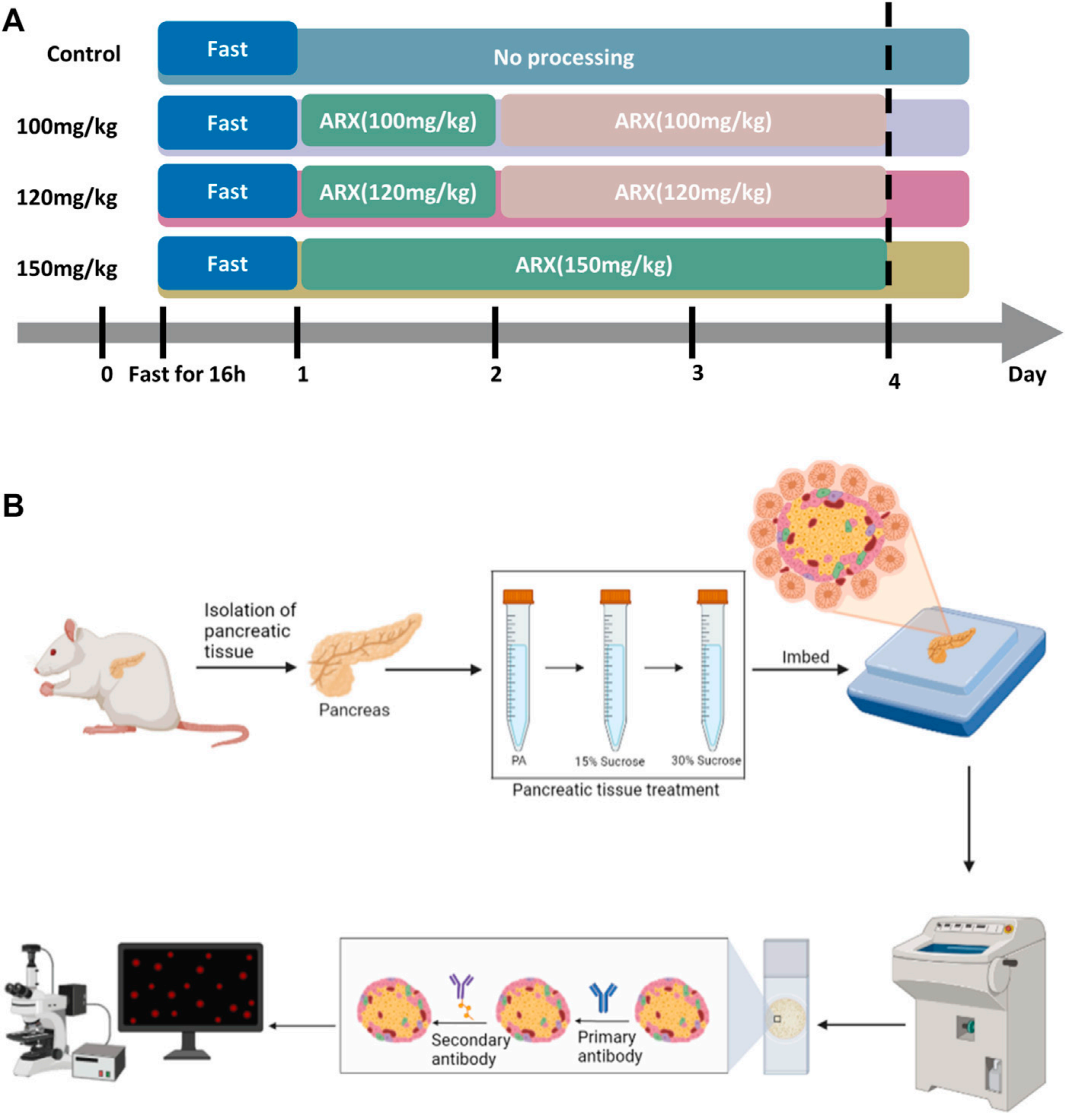
Process of establishing the rat diabetes models and immunofluorescence staining in type 1 diabetic rats. Figure **(A)** the diabetes models with different degrees. Four groups of rats (control, 100 mg/kg, 120 mg/mg, and 150 mg/kg) were injected with the corresponding dose of alloxan solution after 16 h of fasting; the 100 mg/kg and 120 mg/kg groups were injected with the same dose of alloxan solution again 24 h after the first injection, and the control group was not treated. The models were established 72 h after the first injection. Figure **(B)** the immunofluorescence staining procedure. The isolated pancreatic tissue was fixed in paraformaldehyde, dehydrated in a sucrose gradient, and embedded in optimal cutting temperature solution; serial slices were prepared in a frozen slicer, mounted on adhesive slides, the tissue sections were treated with primary and secondary antibodies, and the images were captured under a laser confocal microscope. The immunofluorescence images were processed using software, and quantitative and correlation analyses were performed.

**FIGURE 3:**
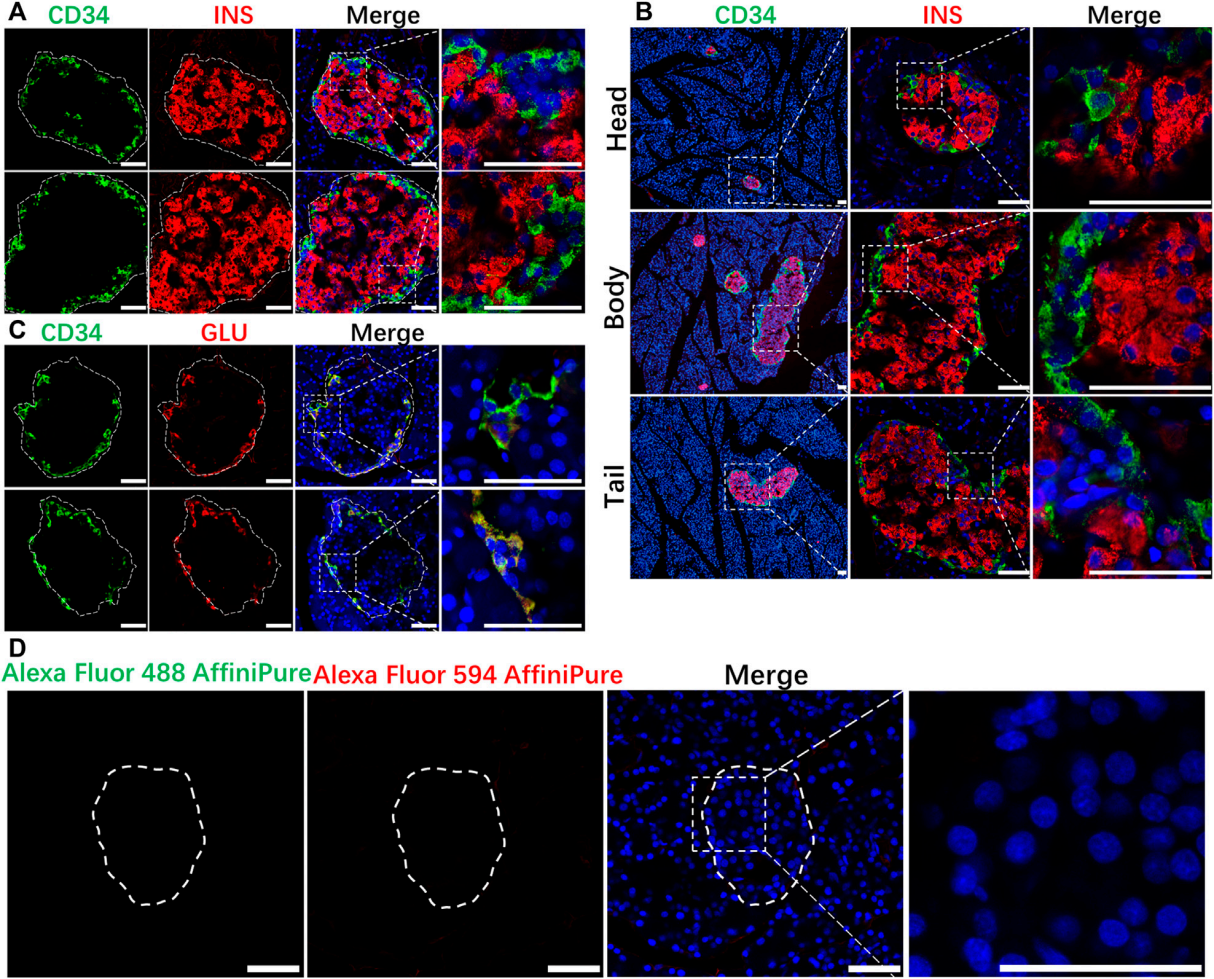
Localization of CD34 in normal islets. Figure **(A)** the relationship between CD34 and Insulin-labeled islet β-cells. Figure **(B)** the location of CD34 in islets in the pancreas head, body, and tail. Figure **(C)** the relationship between CD34 and Glucagon-labeled islet α-cells. Figure **(D)** the images of negative control experiment. Green: CD34; Red: Insulin/Glucagon; Blue: DAPI. The irregular dotted frame is the islet area and the rectangular dotted frame is the area to be magnified. Scale bar = 50 μm.

The present study was mainly carried out according to the procedures in Figure 2. The IF results in normal rat pancreatic tissue showed that CD34 was stably expressed in the periphery of the rat islet structure and was not localized on INS-labeled islet β-cells (Figure 3A). Although the proportion of INS expression differed in pancreatic head, body, tail, the expression of CD34 was detected in all those parts and CD34 in all pancreatic tissue samples were localized in the periphery of islet structure, not in islet β-cells and non-islet cells. (Figure 3B). The negative control results demonstrated the critical localization of CD34 in pancreatic islets. (Figure 3D).

### 3.3 CD34 expression increased significantly on INS-positive cells decreased diabetes model rats

**FIGURE 4:**
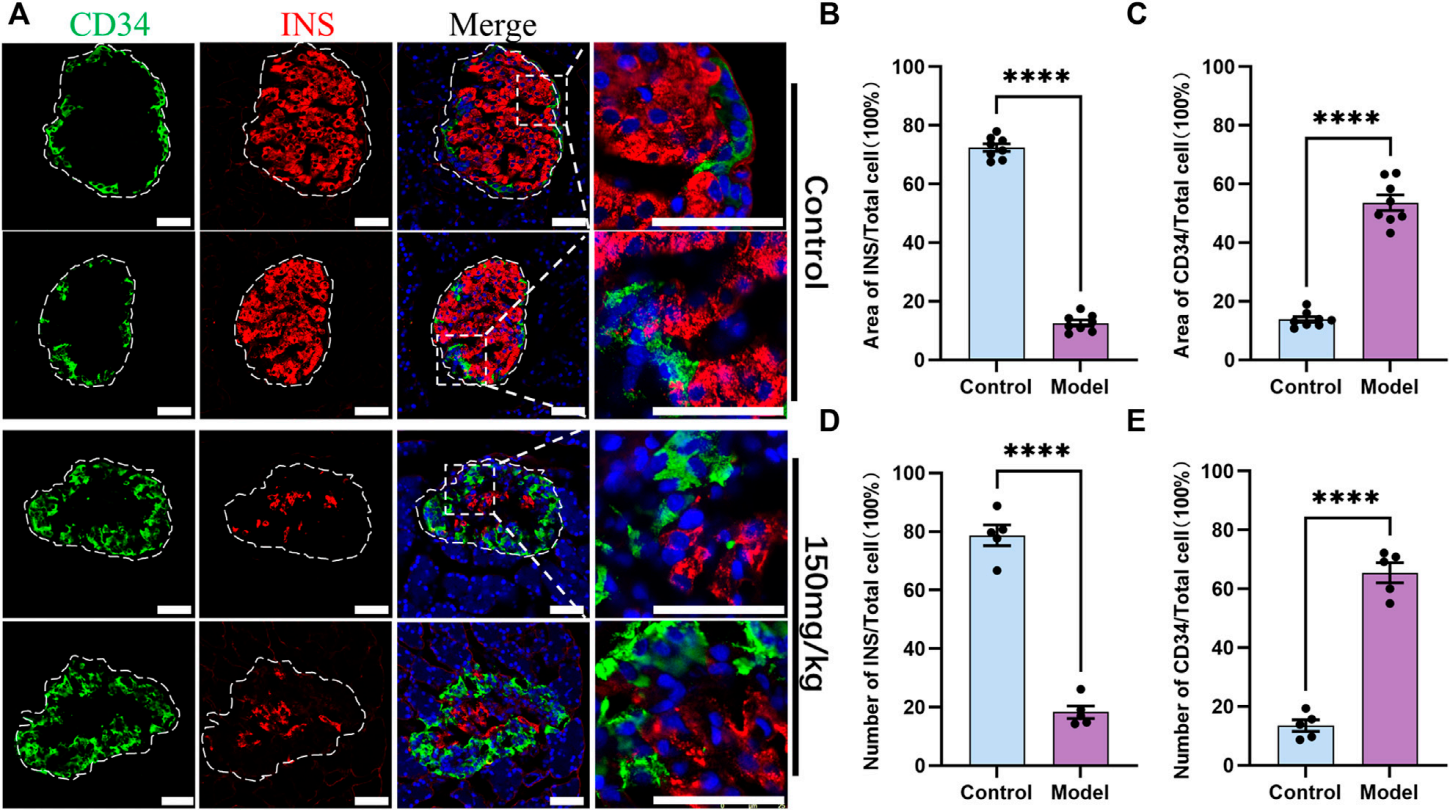
Control and diabetes model (150 mg/kg) rat islets’ immunofluorescence staining and analysis. Figure **(A)** immunofluorescence staining of CD34 and Insulin in the control and diabetes groups. Green: CD34, Red: Insulin; Blue: DAPI. The white irregular dotted frame is the islet area and the white rectangular dashed frame is the enlarged area; scale bar = 50 μm. Figure **(B)** the proportion of Insulin-positive area to the entire islet area between the control and diabetes groups. Figure **(C)** the proportion of CD34-positive area to the entire islet area between the control and diabetes groups. Figure **(D)** the proportion of Insulin-positive cell number to the total islet cell number between the control and diabetes groups. Figure **(E)** the proportion of CD34-positive cell number to the total islet cell number between the control and diabetes groups. The data of each group are presented as mean ± standard error, and the difference was analyzed *via* an independent sample *t*-test. ****p < 0.0001.

IF was then performed on the pancreas sections derived from the rats of the control group and type 1 diabetes model group (150 mg/kg) to explore the relationship between CD34 and islet endocrine cells. The staining results demonstrated that the INS-positive cells were significantly decreased in the model group and the CD34-positive cells were increased (Figure 4A). Compared with the control group, the proportion of INS-positive area in the diabetes model group decreased significantly (from 0.7243 ± 0.0131 to 0.1258 ± 0.0102) (Figure 4B), whereas the proportion of CD34-positive area increased significantly (from 0.1375 ± 0.00923 to 0.5364 ± 0.0266) (Figure 4C). Likewise, the same results were obtained in the cell count, i.e., the proportion of INS-positive cells decreased significantly (from 0.7872 ± 0.0355 to 0.1826 ± 0.0214) (Figure 4D) and the proportion of CD34-positive cells increased significantly (from 0.1348 ± 0.0197 to 0.6546 ± 0.0338) (Figure 4E). In order to eliminate the influence of double-labeled antibody interaction on the experimental results, we used single-labeled INS staining experiment for comparison, and the results were consistent with double-labeling staining (Supplementary Figure S1A). In addition, increased CD34 expression was detected in the original islet β-cell region. These results indicated that there is a negative correlation between the expression of CD34 and damage of β-cells in diabetes.

### 3.4 CD34 consistently colocalized with GLU in islets

**FIGURE 5:**
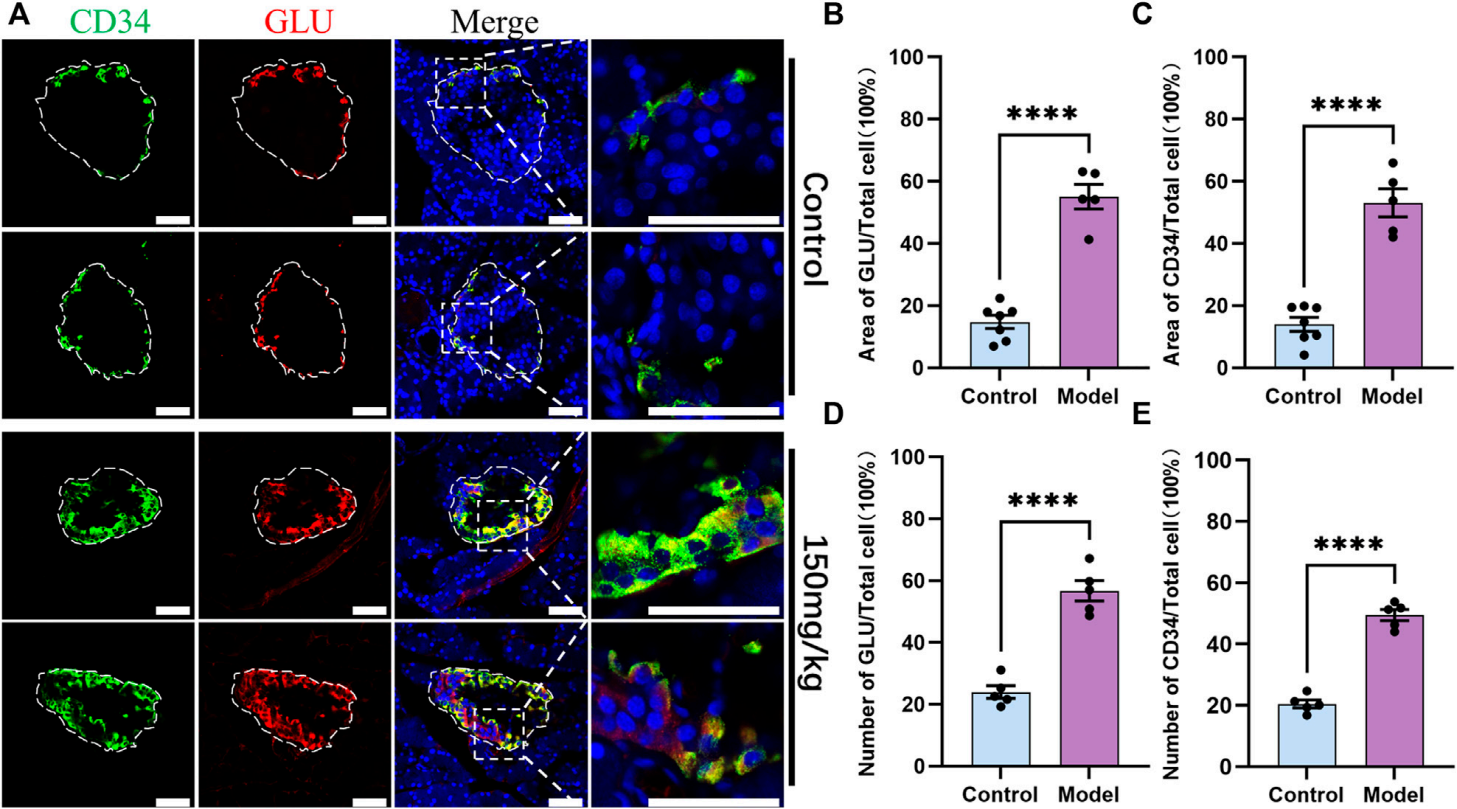
Localization of CD34 and Glucagon in the islets of rats in the control and diabetes groups (150 mg/kg). Figure **(A)** immunofluorescence staining of CD34 and Glucagon in the control and diabetes groups. Green: CD34, Red: Glucagon; Blue: DAPI. The white irregular dotted frame is the islet area and the white rectangular dashed frame is the enlarged area; scale bar = 50 μm. Figure **(B)** the proportion of Glucagon-positive area to the entire islet area between the control and diabetes groups. Figure **(C)** the proportion of CD34-positive area to the entire islet area between the control and diabetes groups. Figure **(D)** the proportion of Glucagon-positive cell number to the total islet cell number between the control and diabetes groups. Figure **(E)** the proportion of CD34-positive cell number to the total islet cell number between the control and diabetes groups. The data of each group are presented as mean ± standard error, and the difference was analyzed *via* an independent sample *t*-test. ****p < 0.0001.

As CD34 was expressed in the periphery of the islet structure, the role of CD34 and GLU-labeled islet α-cells was explored. IF staining results revealed that CD34 and GLU were colocalized in islet α-cells (Figure 3C). CD34 and GLU were highly colocalized in the diabetes model, and their expression in the diabetes model was significantly higher than that in the control group (Figure 5A). Statistical analysis showed that the proportion of GLU-positive area in islets increased from 0.1472 ± 0.0213 to 0.5507 ± 0.0394 (*p* < 0.0001) (Figure 5B), the proportion of CD34-positive area increased from 0.1397 ± 0.0228 to 0.5307 ± 0.0455 (*p* < 0.0001) (Figure 5 C), the proportion of GLU-positive cells increased from 0.2398 ± 0.0202 to 0.5672 ± 0.0330 (*p* < 0.0001) (Figure 5D), and the proportion of CD34-positive cells in islets increased from 0.2046 ± 0.0129 to 0.4944 ± 0.0184 (*p* < 0.0001) (Figure 5E). Similarly, single-labeled GLU staining results were consistent with the results of double-labeled staining (Supplementary Figure S1B).

### 3.5 CD34 expression increased gradually with the progression of diabetes

**FIGURE 6:**
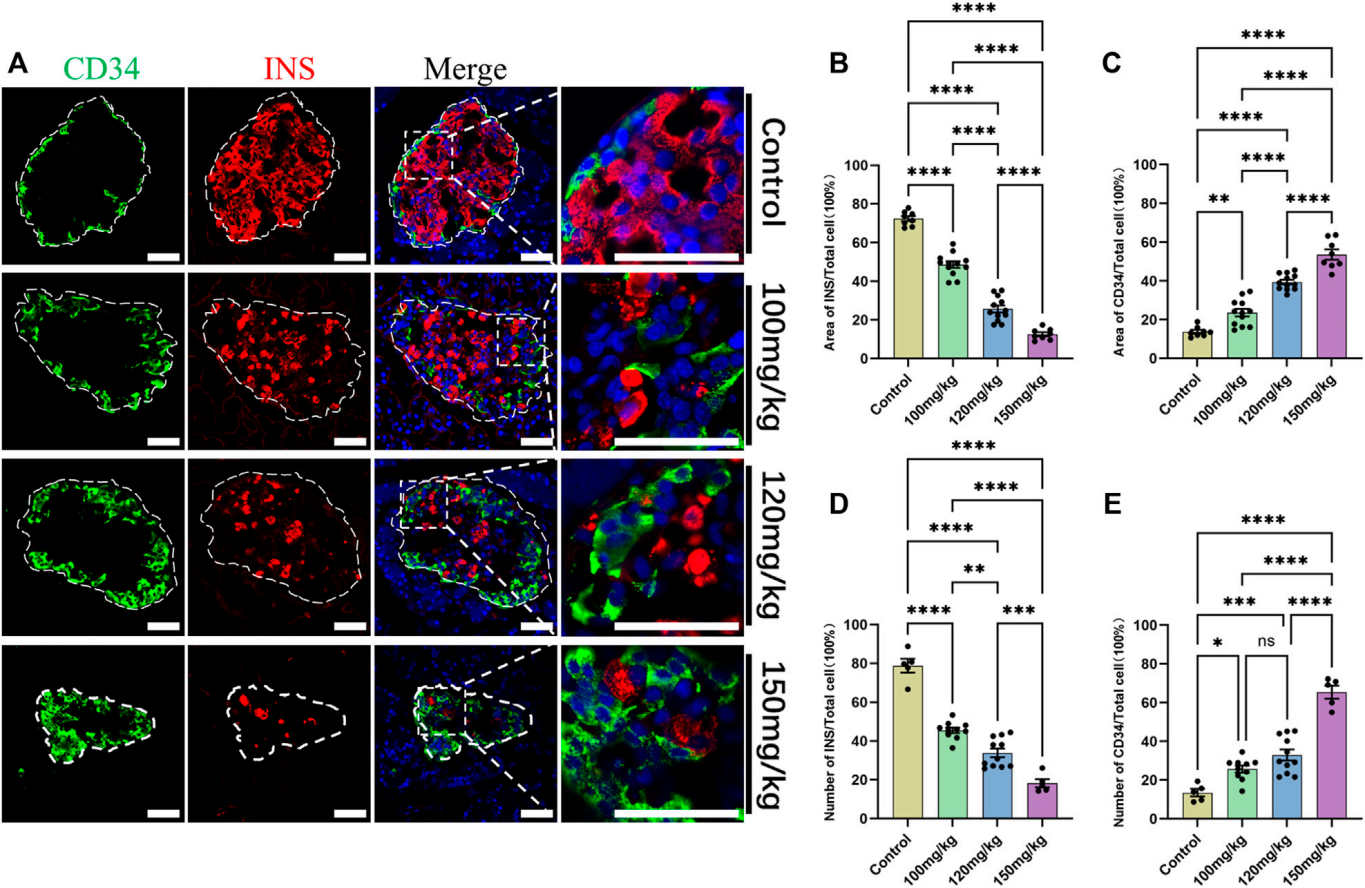
Change in CD34 expression in the progression of diabetes. Figure **(A)** the localization of CD34 in the islets of rats in the control and diabetes model groups established with gradient alloxan (100 mg/kg, 120 mg/kg, and 150 mg/kg). Green: CD34, Red: Insulin; Blue: DAPI. The white irregular dotted frame is the islet area, and the white rectangular dashed frame is the enlarged area; scale bar = 50 μm. Figure **(B)** the percentage of Insulin-positive area to the entire islet region between the control and diabetes groups of different degrees. Figure **(C)** the percentage of CD34-positive area to the entire islet region between the control and diabetes groups of different degrees. Figure **(D)** the percentage of Insulin-positive cell number to the total islet cell number between the control and diabetes groups of different degrees. Figure **(E)** the percentage of CD34-positive cell number to the total islet cell number between the control and diabetes groups of different degrees. *p < 0.05, **p < 0.01, ***p < 0.001, ****p < 0.0001, ns *p* > 0.05. The sample data of each group are presented as mean ± standard error, and the difference was analyzed using one-way ANOVA.

For further verifying the relationship between CD34 and islet β-cell failure in the progression of diabetes, IF staining and statistical data visualization were performed in the pancreas sections of five rats that were dosed with the different concentrations of alloxan. As the concentration of alloxan was increased, the extent of diabetes and damage of islet β-cells increased (Figure 6A) and the number (Figure 6B) and area (Figure 6D) of β-cells were significantly reduced. Therefore, diabetic model induced by different concentrations of alloxan can simulate the islet function state at different times during the progression of diabetes with varying degrees. Compared with the control group, the expression of CD34 increased gradually with the degree of islet β-cell damage in the rats dosed with the different concentrations of alloxan (Figure 6A). The proportion of number (Figure 6C) and area (Figure 6E) of CD34-positive labeled cells are gradually increasing as the degree of diabetes progresses.

**FIGURE 7:**
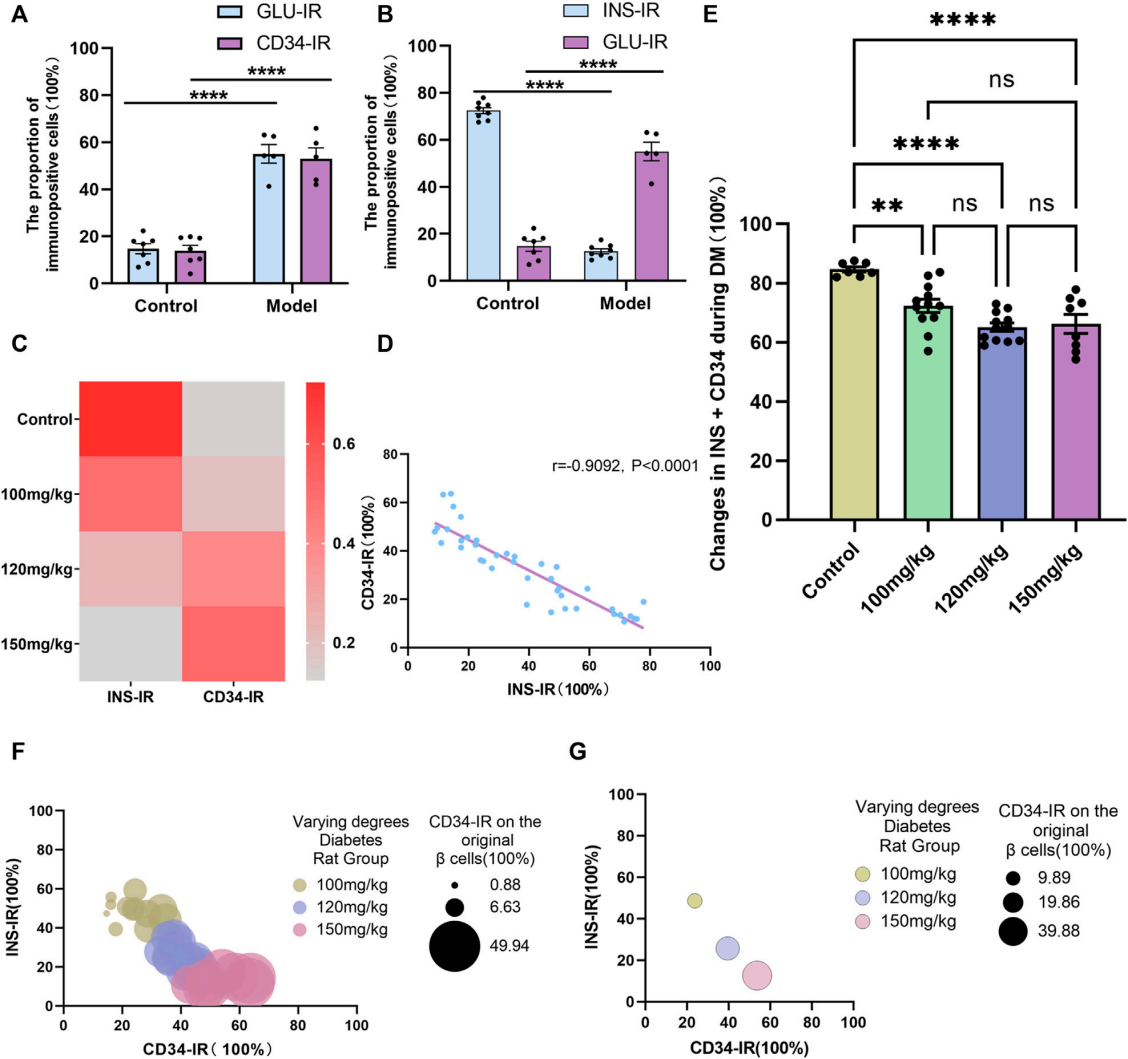
Linear and negative correlation between CD34 and islet β-cells. Figure **(A)** Analysis of the difference in Glucagon and CD34 expression between the control and model groups. Figure **(B)** comparison of the proportion of Insulin- and Glucagon-positive expression to the islet structure in the control group/model group. Figure **(C)** the relationship between Insulin- and CD34-positive expression between the normal group and different degree diabetes groups. Figure **(D)** linear correlation analysis of the proportion of CD34-positive area and Insulin-positive area in the progression of diabetes. Figure **(E)** analysis of the proportion of CD34-positive area + Insulin-positive area in the progression of diabetes. Figure **(F)** the relationship among the proportion of CD34-positive area, Insulin-positive area, and proportion of CD34-positive on islet β-cells in diabetes. Figure **(G)** the relationship between CD34-positive area and the proportion of Insulin-positive region, and the proportion of CD34 and β-cells in different degree diabetes model groups. *p < 0.05 denotes statistically significant.

Of note, there was no difference between the GLU- and CD34-labeled areas/cells before and after diabetes (Figure 7A); however, compared with the control group (Figure 7B, C), the INS-positive localization areas decreased significantly and GLU-positive localization areas increased significantly in the diabetes model group (*p* < 0.0001), and CD34 was expressed in the distribution region of former islet β-cells in relative Control group. These results suggested that CD34 plays the same role as GLU in labeling islet α-cells and that some islet β-cells may transdifferentiate into islet α-cells during diabetes, which can be identified using CD34.

### 3.6 CD34 was negatively correlated with the number of islets β-cell

Additional analysis of the experimental data showed no difference between the CD34^−^ and GLU-labeled regions/cells (Figure 7A). However, in anticipation of further exploring the relationship between CD34^−^ and INS-labeled islet β-cells, linear regression analysis (linear regression) on the staining data of pancreatic sections from each group was performed. The results revealed that as diabetes progressed, a significant negative correlation was observed between changes in the CD34-positive localization region and INS-positive localization region in the islet structure (r = −0.9092, *p* < 0.0001) (Figure 7D). These results strongly demonstrated the adequacy of CD34 as a β-cell biomarker, i.e., changes in CD34 expression can indicate the state of β-cell failure in islets.

Further quantitative analysis revealed that compared with the control group, the proportion of the sum of INS-positive and CD34-positive regions in the islet region of each degree of the diabetes models decreased to a certain extent (Figure 7E), which occurred because of β-cell failure in the occurrence and development of diabetes. Of note, there was no difference in the proportion of INS-positive and CD34-positive regions in the islet region among the different degrees of the diabetes models. Moreover, with the progression of diabetes, the expression of CD34 in islets increased and the proportion of expression in the original β-cells increased (Figure 7F, G). These results also indirectly indicated the possible of islet β-cell transdifferentiation in diabetic islets.

## 4 Discussion

To the best of our knowledge, the present study for the first time indicated that CD34 is specifically localized on rat islet α-cells and higher CD34 expression can predict reduced β-cell number in pancreatic islets, which may be utilized as a biomarker of islet β-cell in the early diagnosis of diabetes. Furthermore, increased CD34-positive α-cells in the pancreatic islets of diabetes models indicated that some β-cell may undergo transdifferentiation rather than apoptosis in the progression of diabetes and also lose the function of INS secretion.

CD34 is used as a biomarker of hematopoietic stem cells and hematopoietic progenitor cells (Nielsen and McNagny, 2008) and has been predicted to be a biomarker of certain other nonhematopoietic cell types, such as vascular endothelial progenitor cells and embryonic fibroblasts (Krause et al., 1996). Research has shown that CD34, as a structurally stable antigen, can be expressed on a variety of cells (Deterding et al., 2011). For example, CD34 is widely expressed on pluripotent mesenchymal stromal cells, interstitial dendritic cells, and epithelial progenitor cells (Nakayama et al., 2000; Blanpain et al., 2004; Lin et al., 2012), and studies have shown that CD34 and other CD family proteins play a critical role in pancreatic endocrine islets (Merkwitz et al., 2011; Kobayashi et al., 2018). In this study, the biomarker function of CD34, which has been widely used for other cells, was evaluated for islet β-cells during diabetes.

Based on the ProteomicsDB analysis, CD34 was expressed consistently in mouse and human pancreatic tissues. In addition, CD34 was exclusively expressed on islet α-cells but not on INS-labeled islet β-cells, which was consistent with one report on fetal cattle (Merkwitz et al., 2011). Contrary to previous reports (Merkwitz et al., 2011), the present study newly showed that CD34 is stably expressed on adult rat islet α-cells and the expression does not reduce compared with the maturation of islet α-cells.

The diabetes models showed that CD34 has the potential to be used as a biomarker for monitoring β-cell failure in the progression of diabetes, i.e., a strong linear and negative correlation exists between the loss of β-cells and increase of CD34 cells during disease progression. Furthermore, the proportion of CD34 and GLU in islet α-cells was significantly higher than that in the control, and changes in the islet α-cells observed in the present study is consistent with previous reports (Plesner et al., 2014; Titova et al., 2020), suggesting that some islet β-cells did not undergo apoptosis but lost the function of INS secretion and differentiated into GLU-positive islet α-cells, these transdifferentiated islet β-cells could be labeled by CD34. Interestingly, our experimental on the relationship between islet α-cells and β-cells during diabetes also suggested that transdifferentiation of islet β-cells into α-cells may be occur during diabetes. These results are inconsistent with the report on the destruction of islet β-cells in type 1 diabetes, resulting in the absolute lack of INS production (Syed, 2022), but consistent with the report on the transformation of the identity of islet endocrine cells in type 2 diabetes (Moin and Butler, 2019). This suggests that in type 1 diabetes, islet β-cell transdifferentiation is involved in the loss of INS secretion function. Although CD34 can be used as a biomarker of transdifferentiated islet β-cell, it cannot further distinguish between the original islet α-cell and an islet α-cell that transdifferentiated from an islet β-cell. Therefore, it may be necessary to further combine islet β-cell-specific expression proteins (such as the β-cell-specific transcription factor homeobox protein Nkx6.1) to identify such β-cells that transdifferentiated (Aigha and Abdelalim, 2020). An interesting phenomenon is that the transdifferentiated islet β-cell always extends gradually from the periphery to the center. This phenomenon of islet structural remodeling may involve islet cell–cell, islet cell–matrix, or changes in cell composition and paracrine signals between islet cells (Spijker et al., 2013), which has not been evaluated here. In the process of islet β-cell injury induced by alloxan, islet β-cells lose INS secretion function and dedifferentiate into undifferentiated stem cells or progenitor cells, which can be labeled by CD34, and eventually transform into islet α-cells. In addition, several studies have shown that CD34 has the function of regulating cell proliferation and differentiation (Cheng et al., 1996; Krause et al., 1996; Nielsen and McNagny, 2008). Considering the study results, CD34 also possibly plays a promoting role in the production and secretion of GLU. The role of CD34 in islet β-cell transdifferentiation and the reason for its specific expression on islet α-cells remains unclear and is worthy of further exploration. The results also suggest that islet β-cell apoptosis and islet β-cell transdifferentiation mediate the insufficient secretion of INS in the development of alloxan-induced type 1 diabetes.

**FIGURE 8:**
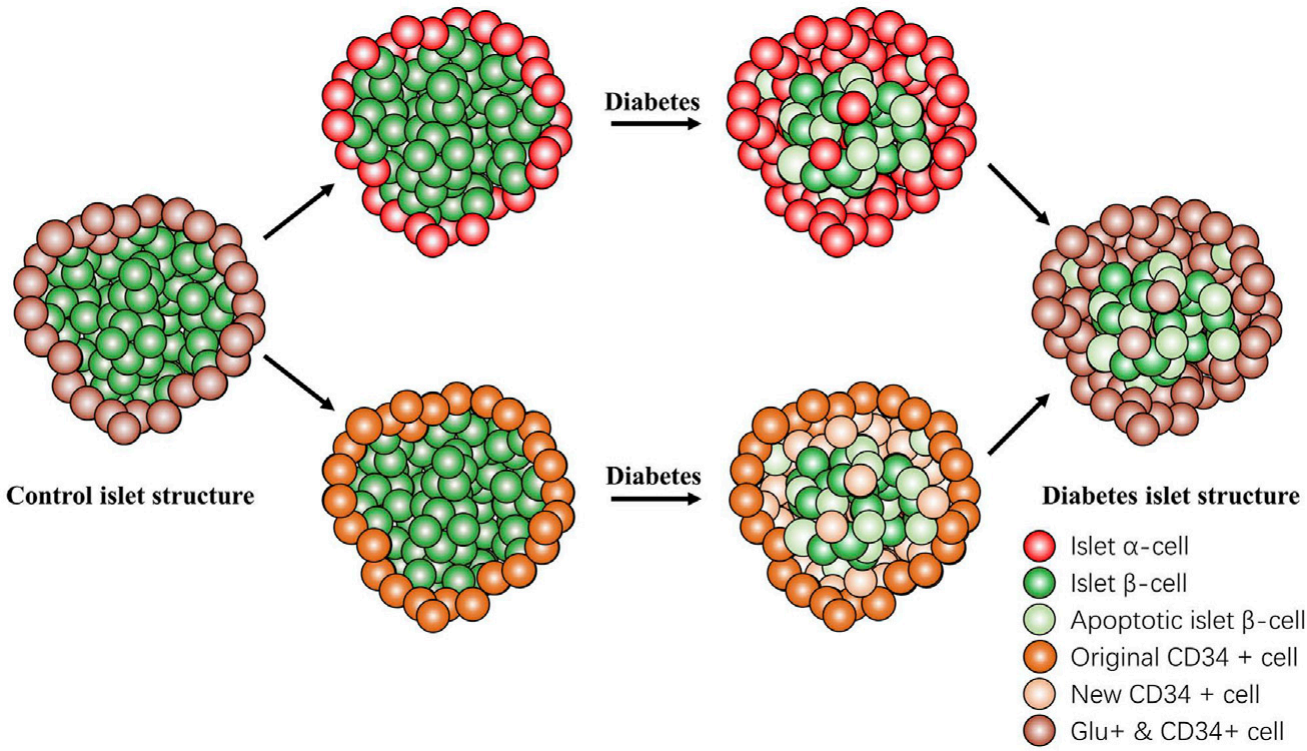
Changes in the islet structure in diabetes. In the progression of diabetes, the number of normal islet β-cells gradually decreased and that of CD34-positive cells gradually increased. The newly CD34-expressing cells were originally islet β-cells that were transdifferentiated and lost the function of secreting insulin but did not undergo apoptosis and transformed into glucagon-positive and glucagon-secreting islet α-cell. CD34 and glucagon always coexist in the same cell. Islet β-cell apoptosis and islet β-cell transdifferentiation participate in the failure of islet β-cell function. Red: Islet α-cell; green: Islet β-cell; light green: apoptotic islet β-cell; orange: original CD34^+^ cell; light orange: new CD34^+^ cell; and brown: Glucagon+ & CD34^+^ cell.

In conclusion, the present study demonstrated that CD34 is expressed on GLU-secreting islet α-cells but not on INS-secreting islet β-cells. The progression of diabetes can be predicted by monitoring the expression of CD34 in the islet structure of patients with diabetes. Furthermore, CD34 may be used to identify transdifferentiated β-cells; however, it is not yet possible to distinguish between original islet α-cells and from β-cell-transformed islet α-cells (Figure 8). The present study findings may provide new candidate targets for islet β-cells in diabetes diagnosis and for predicting the newly identified transdifferentiation mechanism in diabetes.

## Data Availability

The original contributions presented in the study are included in the article/Supplementary Materials, further inquiries can be directed to the corresponding authors.

## Supplementary material

Supplementary Figure S1 Comparative experimental results of single immunofluorescence staining. Figure **(A)** Experimental results of single insulin staining; Figure **(B)** Experimental results of single glucagon staining; Green: CD34, Red: Insulin or Glucagon; Blue: DAPI. The white irregular dotted frame is the islet area; scale bar = 20 μm.
