# Supplementary figures and images for: Increased CD34 in pancreatic islet negatively predict islet β-cell decrease in type1 diabetes model
Source: Front Physiol. 2022 Nov 18;13:1032774. doi: 10.3389/fphys.2022.1032774 (PMC9716098; doi:10.3389/fphys.2022.1032774)

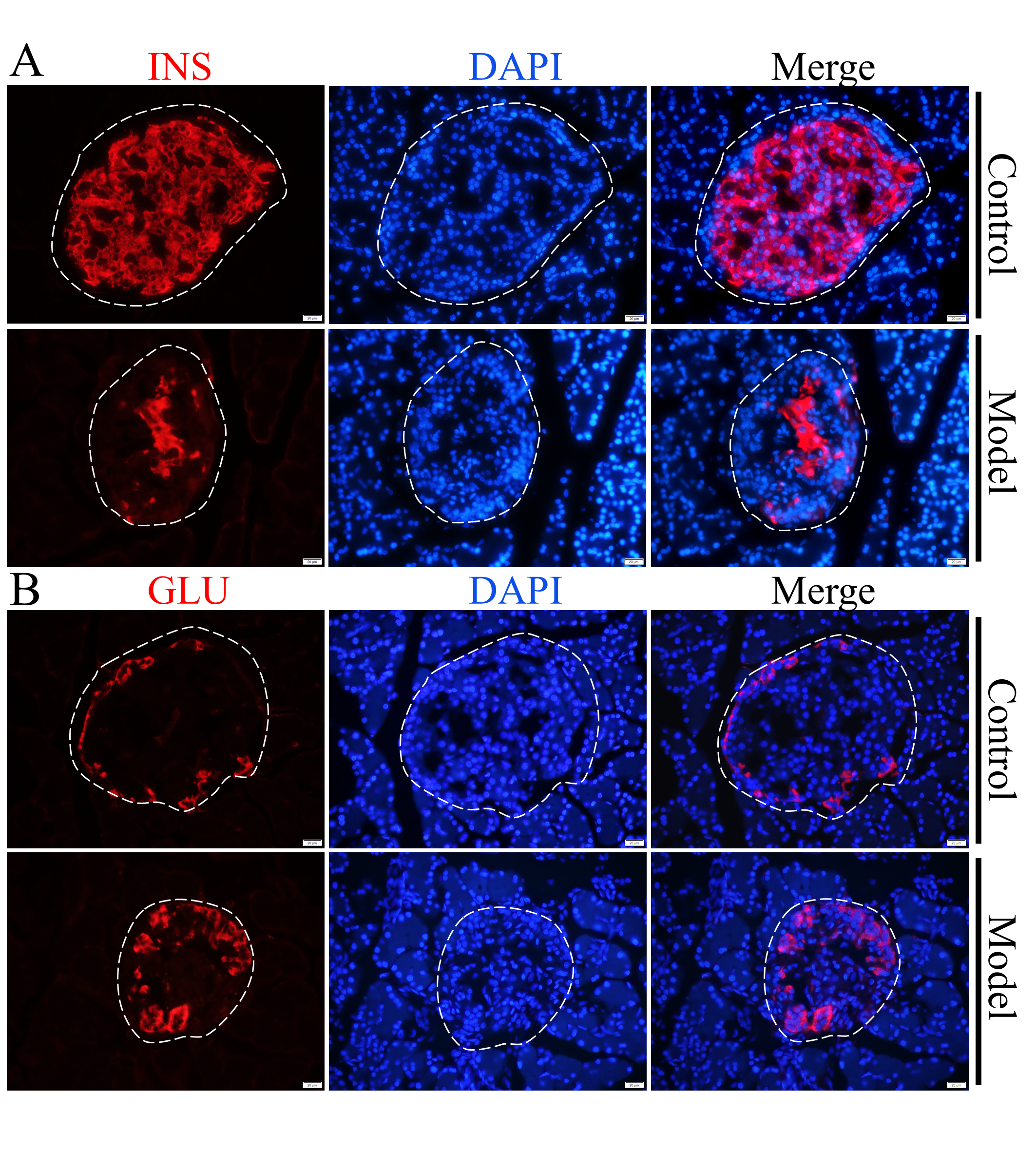

Supplement: Supplementary file 2 [file Image1.TIF]
